# Large-scale genomic prediction using singular value decomposition of the genotype matrix

**DOI:** 10.1186/s12711-018-0373-2

**Published:** 2018-02-28

**Authors:** Jørgen Ødegård, Ulf Indahl, Ismo Strandén, Theo H. E. Meuwissen

**Affiliations:** 1 AquaGen AS, P.O. Box 1240, 7462 Trondheim, Norway; 2 Norwegian University of Life Sciences, P.O. Box 5003, 1432 Ås, Norway; 3 Natural Resources Institute Finland (Luke), Humppilantie 14, Jokioinen, Finland

## Abstract

**Background:**

For marker effect models and genomic animal models, computational requirements increase with the number of loci and the number of genotyped individuals, respectively. In the latter case, the inverse genomic relationship matrix (GRM) is typically needed, which is computationally demanding to compute for large datasets. Thus, there is a great need for dimensionality-reduction methods that can analyze massive genomic data. For this purpose, we developed reduced-dimension singular value decomposition (SVD) based models for genomic prediction.

**Methods:**

Fast SVD is performed by analyzing different chromosomes/genome segments in parallel and/or by restricting SVD to a limited core of genotyped individuals, producing chromosome- or segment-specific principal components (PC). Given a limited effective population size, nearly all the genetic variation can be effectively captured by a limited number of PC. Genomic prediction can then be performed either by PC ridge regression (PCRR) or by genomic animal models using an inverse GRM computed from the chosen PC (PCIG). In the latter case, computation of the inverse GRM will be feasible for any number of genotyped individuals and can be readily produced row- or element-wise.

**Results:**

Using simulated data, we show that PCRR and PCIG models, using chromosome-wise SVD of a core sample of individuals, are appropriate for genomic prediction in a larger population, and results in virtually identical predicted breeding values as the original full-dimension genomic model (r = 1.000). Compared with other algorithms (e.g. algorithm for proven and young animals, APY), the (chromosome-wise SVD-based) PCRR and PCIG models were more robust to size of the core sample, giving nearly identical results even down to 500 core individuals. The method was also successfully tested on a large multi-breed dataset.

**Conclusions:**

SVD can be used for dimensionality reduction of large genomic datasets. After SVD, genomic prediction using dense genomic data and many genotyped individuals can be done in a computationally efficient manner. Using this method, the resulting genomic estimated breeding values were virtually identical to those computed from a full-dimension genomic model.

**Electronic supplementary material:**

The online version of this article (10.1186/s12711-018-0373-2) contains supplementary material, which is available to authorized users.

## Background

In recent years, genomic prediction [1] has revolutionized animal and plant breeding methods. With decreasing genotyping costs, the number of genotyped individuals has increased exponentially over years, with up to full sequence of genomic information available for prediction. Genomic prediction can be performed using two families of genomic models: marker effects models (MEM) (e.g. SNP-best linear unbiased prediction (BLUP), BayesA, BayesB, BayesC, etc.), and animal models that use a genomic relationship matrix (GRM). The latter can be further divided into genomic models that include genotyped animals only (genomic BLUP, i.e. GBLUP) and single-step GBLUP (ssGBLUP) models [2, 3] that combine genotyped and ungenotyped animals. The advantage of genomic animal models is that they fit nicely within the traditional linear models’ framework, and can essentially be adapted to any kind of linear or generalized linear animal model (single-trait, multi-trait, random regression, etc.).

However, with the increasing number of genotyped individuals and increasing density of genotypes, the computational requirements of genomic prediction models increase accordingly. Hence, MEM analysis of full sequence data, e.g. using Bayesian variable selection models, will be very demanding in terms of computing time. For ssGBLUP [2, 3], the inverse of the GRM is computed prior to analysis, which may be practically impossible when the number of genotyped animals becomes very large (e.g. > 100,000). To address the latter, Misztal et al. [4] proposed the “algorithm for proven and young animals” (APY), which uses a core sample of individuals to compute an approximate inverse of the GRM for all animals. However, in some cases, the total GRM does not have full rank, and thus no inverse. Therefore, Fernando et al. [5] suggested exact methods to obtain ssGBLUP solutions. One of the options that they proposed was to model animal genetic effects as linear combinations of independent factors. In the following section, we propose a related strategy that applies singular value decomposition (SVD) to perform large-scale genomic evaluation, both for MEM and animal genomic models. Thus, our study aims at: (1) using SVD and principal component (PC) ridge regression (PCRR) for genomic prediction as an alternative to MEM, using up to full sequence genomic data, and (2) applying SVD techniques for computation of exact inverses of PC-based GRM, using dimensionality reduction.

## Methods

### Marker effect models

Assume that dense single nucleotide polymorphism (SNP) genotypes for $k$ loci are available for $N$ animals. Omitting fixed effects for simplicity, the simplest MEM (called SNP-BLUP) can be specified as [1]:

1 $${\mathbf{y}} = {\mathbf{Xb}} + {\mathbf{e}},$$

where ${\mathbf{y}}$ is a vector of phenotypes, ${\mathbf{X}}$ is an $N \times k$ (centered) matrix of genotype dosage for all SNPs and all animals, ${\mathbf{b}} \sim N\left( {{\mathbf{0}},{\mathbf{I}}\sigma_{m}^{2} } \right)$ is a vector of SNP allele substitution effects, $\sigma_{m}^{2}$ is the variance of SNP effects, ${\mathbf{e}} \sim N\left( {{\mathbf{0}},{\mathbf{I}}\sigma_{e}^{2} } \right)$ is a vector of random residuals, and $\sigma_{e}^{2}$ is the residual variance. The SNP-BLUP equations [6] are:

2 $$\left[ {{\mathbf{X}^{\prime}\mathbf{X}} + \lambda {\mathbf{I}}} \right]{\hat{\mathbf{b}}} = {\mathbf{X}}'{\mathbf{y}},$$

where $\lambda = \frac{{\sigma_{e}^{2} }}{{\sigma_{m}^{2} }}$ is the ratio of residual variance to SNP effects variance. Here, we assume that the SNP effects variance is: $\sigma_{m}^{2} = \frac{{\sigma_{g}^{2} }}{{2\mathop \sum \nolimits_{i = 1}^{k} p_{i} \left( {1 - p_{i} } \right)}}$, where $\sigma_{g}^{2}$ is the total additive genetic variance and $p_{i}$ is the allele frequency at locus $i$. The dimension of the equation system is equal to the number of loci ($k$). Hence, if $k$ is large (e.g. full sequence), solving this system of equations may be difficult.

### Gblup

A GBLUP (animal) model, equivalent to the above SNP-BLUP model (i.e. assuming all animals have data) is [7]:

3 $${\mathbf{y}} = {\mathbf{g}} + {\mathbf{e}},$$

where ${\mathbf{e}}$ is as defined above and ${\mathbf{g}} \sim N\left( {{\mathbf{0}},{\mathbf{G}}\sigma_{g}^{2} } \right)$ is a vector of additive genetic effects. Now, the equation system becomes [7]:

4 $$\left[ {{\mathbf{I}} + \lambda_{g} {\mathbf{G}}^{ - 1} } \right]{\hat{\mathbf{g}}} = {\mathbf{y}},$$

where $\lambda = \frac{{\sigma_{e}^{2} }}{{\sigma_{g}^{2} }}$, i.e. the ratio of residual to total additive genetic variance. The GRM ${\mathbf{G}}$ is a function of the observed genotypes, e.g. based on VanRaden’s Method 1 [8], ${\mathbf{G}} = \frac{1}{\rho }{\mathbf{XX}}'$, where $\rho = 2{\mathbf{p}}'\left( {1 - {\mathbf{p}}} \right)$, with ${\mathbf{p}}$ being a vector of SNP allele frequencies in the population. In populations of limited effective size ($N_{e}$), the genomic relationships are a result of the segregation of a limited number of haplotype segments, and thus, ${\mathbf{G}}$ may not be positive definite, implying that its inverse does not exist. In such cases, ${\mathbf{G}}$ is still positive semidefinite, i.e., for any non-zero vector ${\mathbf{z}}$ of *N* real numbers: ${\mathbf{z^{\prime}Gz}} = \frac{1}{\rho }{\mathbf{z^{\prime}XX^{\prime}z}} = \frac{1}{\rho }{\mathbf{u^{\prime}u}} \ge 0$ $\left( {{\mathbf{u}} = {\mathbf{X^{\prime}z}}} \right)$. We defined an approximated GRM: ${\tilde{\mathbf{G}}} = \left( {{\mathbf{G}} + {\mathbf{I}}\theta } \right) = \frac{1}{\rho }\left( {{\mathbf{XX^{\prime}}} + {\mathbf{I}}\rho \theta } \right)$, where $\theta$ is a small number (e.g. 10^−3^). The matrix ${\tilde{\mathbf{G}}}$ is positive definite, and thus invertible, as: ${\mathbf{z}^{\prime}\tilde{\mathbf{G}}\mathbf{z}} = \frac{1}{\rho } \cdot {\mathbf{u^{\prime}u}} + \theta \cdot {\mathbf{z^{\prime}z}} > 0$. Adding $\theta$ to the GRM diagonal elements has a negligible effect on the solutions and may be viewed as fitting a (tiny) fraction of the residual as a part of the additive genetic effects, and thus is essentially equivalent to the original GBLUP model. Although ${\tilde{\mathbf{G}}}^{ - 1}$ exists, computing it by direct “brute-force” inversion will be increasingly challenging, and eventually impossible, as the number of genotyped individuals increases (e.g. for $N$ > 100,000). Another option is to specify the equation system as [9]:

5 $$\left[ {{\mathbf{G}} + \lambda_{g} {\mathbf{I}}} \right]{\hat{\mathbf{g}}} = {\mathbf{Gy}},$$

which do not require an invertible ${\mathbf{G}}$. However, for typical single-step evaluations, the inverse of the GRM is still needed [2, 3]. The dimension of the GRM is equal to number of genotyped animals $N$, which may be smaller than number of loci $k$ (at least for dense genomic data). In the opposite case, when the number of genotyped animals exceeds the number of loci, ${\mathbf{G}}$ does not exist, but the exact inverse of ${\tilde{\mathbf{G}}}$ can be calculated by the Woodbury formula [10]:

6 $$\begin{aligned} {\tilde{\mathbf{G}}}^{ - 1} & = \rho \left( {{\mathbf{XX^{\prime}}} + {\mathbf{I}}\rho \theta } \right)^{ - 1} \\ & = \rho \left( {{\mathbf{I}}\rho^{ - 1} \theta^{ - 1} - {\mathbf{I}}\rho^{ - 1} \theta^{ - 1} {\mathbf{X}}\left( {{\mathbf{I}}_{k} + {\mathbf{X^{\prime}I}}\rho^{ - 1} \theta^{ - 1} {\mathbf{X}}} \right)^{ - 1} {\mathbf{X}}'{\mathbf{I}}\rho^{ - 1} \theta^{ - 1} } \right) \\ & = \frac{1}{\theta }\left( {{\mathbf{I}} - {\mathbf{X}}\left( {{\mathbf{X^{\prime}X}} + {\mathbf{I}}_{k} \rho \theta } \right)^{ - 1} {\mathbf{X^{\prime}}}} \right), \\ \end{aligned}$$

where ${\mathbf{I}}$ and ${\mathbf{I}}_{k}$ are identity matrices of rank $N$ (animals), and $k$ (number of loci), respectively. This implies that the ($k \times k$) matrix $\left( {{\mathbf{X^{\prime}X}} + {\mathbf{I}}_{{\mathbf{k}}} \rho \theta } \right)$ has to be inverted rather than the ($N \times N$) GRM. Still, for large $k$, computing a direct inverse may be computationally difficult. We will show later how dimensionality reduction of genomic data can be used for the efficient computation of the inverse of large GRM.

### Principal component ridge regression (PCRR)

Related animals typically share large segments of DNA, and for dense genomic data, substantial linkage disequilibrium (LD) is expected between closely linked loci. Hence, the genomic variation, even with up to full sequence data, is likely largely explained by a smaller number of underlying components, i.e., using principal component analysis, majority of the genomic variation can be described by a limited number of principal components (PC). For a genotype matrix ${\mathbf{X}}$ of size ($N \times k$), assuming that $N < k$ (more markers than individuals), an economy-sized (i.e. only keeping PC with eigenvalues > 0) SVD e.g. [11] would be:

7 $${\mathbf{X}} = {\mathbf{USV}}',$$

where ${\mathbf{U}}$ is $\left( {N \times N} \right)$, ${\mathbf{V}}$ is $\left( {N \times k} \right)$, and ${\mathbf{S}}$ is a diagonal matrix of dimension $N$, with singular values on the diagonal (square root of eigenvalues). Furthermore, ${\mathbf{U^{\prime}U}} = {\mathbf{UU}}' = {\mathbf{I}}$, and ${\mathbf{V^{\prime}V}} = {\mathbf{I}}$, while ${\mathbf{VV}}' \ne {\mathbf{I}}$ (for $N < k$). The SVD (rectangular matrices) and eigenvalue decomposition (symmetric matrices) have previously been used in genomic models [12, 13]. The SNP-BLUP model can be re-parametrized into a PCRR model [13] by defining ${\mathbf{s}} = {\mathbf{V}}'{\mathbf{b}}$ (PC regression coefficients). The model can be specified as:

8 $${\mathbf{y}} = {\mathbf{Ts}} + {\mathbf{e}},$$

where ${\mathbf{e}}$ is as defined above, the score matrix ${\mathbf{T}} = {\mathbf{US}} = {\mathbf{XV}}$, and ${\mathbf{s}} \sim N\left( {{\mathbf{0}},{\mathbf{I}}\sigma_{m}^{2} } \right)$. There is an exact relationship between solutions to Henderson’s mixed model equations (HMME) that correspond to the PCRR and MEM models, given as ${\hat{\mathbf{b}}} = {\mathbf{V}\hat{\mathbf{s}}}$ [14]. As ${\hat{\mathbf{s}}} = {\mathbf{V}}'{\hat{\mathbf{b}}}$, this implies that ${\hat{\mathbf{b}}} = {\mathbf{VV}}'{\hat{\mathbf{b}}}$, even when ${\mathbf{VV^{\prime}}} \ne {\mathbf{I}}$ (for example when the number of loci exceeds the number of animals), a proof of which is in the Appendix. We illustrate this with the following small numerical example.

Consider centered genotypes of four individuals with five loci as:

$${\mathbf{X}} = \left[ {\begin{array}{*{20}c} 0 & 1 & 0 & 1 & 0 \\ 1 & { - \,1} & { - \,1} & 1 & 0 \\ { - \,1} & 1 & 1 & { - \,1} & 0 \\ 0 & 0 & { - \,1} & { - \,1} & 1 \\ \end{array} } \right],$$

which has more loci than animals. In addition, the genotypes of the four animals are not linearly independent, yielding a genotype matrix of rank 3. Assume that the four individuals have the following phenotypes: $${\mathbf{y}} = \left[ {\begin{array}{*{20}c} {\begin{array}{*{20}c} { - \,0.5} \\ { - \,0.5} \\ \end{array} } \\ {\begin{array}{*{20}c} {0.0} \\ {1.0} \\ \end{array} } \\ \end{array} } \right]$$. Then, using $\lambda = 1$ gives the following SNP effect solutions: $${\hat{\mathbf{b}}} = \left[ {\begin{array}{*{20}c} {\begin{array}{*{20}c} { - \,0.0556} \\ { - \,0.3535} \\ \end{array} } \\ {\begin{array}{*{20}c} { - \,0.1717} \\ { - \,0.3737} \\ {0.2273} \\ \end{array} } \\ \end{array} } \right].$$

As ${\mathbf{X}}$ has rank 3, it can be decomposed as ${\mathbf{X}} = {\mathbf{USV}}\varvec{'}$, keeping the first three components. The SVD matrices of ${\mathbf{X}}$ are:

$${\mathbf{U}} = \left[ {\begin{array}{*{20}c} {0.000000} & {0.525731} & { - \,0.850651} \\ {0.707107} & {0.000000} & {0.000000} \\ { - \,0.707107} & {0.000000} & {0.000000} \\ {0.000000} & { - \,0.850651} & { - \,0.525731} \\ \end{array} } \right],$$

$${\mathbf{S}} = diag\left[ {\begin{array}{*{20}c} {2.82843} \\ {1.90211} \\ {1.17557} \\ \end{array} } \right],\;\;{\text{and}}$$

$${\mathbf{V}} = \left[ {\begin{array}{*{20}c} {0.500000} & {0.000000} & {0.000000} \\ { - \,0.500000} & {0.276393} & { - \,0.723607} \\ { - \,0.500000} & {0.447214} & {0.447214} \\ {0.500000} & {0.723607} & { - \,0.276393} \\ {0.000000} & { - \,0.447214} & { - \,0.447214} \\ \end{array} } \right].$$

$${\text{Then}},\quad {\mathbf{VV^{\prime}}} = \left[ {\begin{array}{*{20}c} {0.25} & { - \,0.25} & { - \,0.25} & {0.25} & {0.00} \\ { - \,0.25} & {0.85} & {0.05} & {0.15} & {0.20} \\ { - \,0.25} & {0.05} & {0.65} & { - \,0.05} & { - \,0.40} \\ {0.25} & {0.15} & { - \,0.05} & {0.85} & { - \,0.20} \\ {0.00} & {0.20} & { - \,0.40} & { - \,0.20} & {0.40} \\ \end{array} } \right],$$

$${\text{and}}\quad {\mathbf{VV}^{\prime}\hat{\mathbf{b}}} = \left[ {\begin{array}{*{20}c} {\begin{array}{*{20}c} { - \,0.0556} \\ { - \,0.3535} \\ \end{array} } \\ {\begin{array}{*{20}c} { - \,0.1717} \\ { - \,0.3737} \\ {0.2273} \\ \end{array} } \\ \end{array} } \right].$$

Hence, $${\mathbf{VV}^{\prime}\hat{\mathbf{b}}} = {\hat{\mathbf{b}}}$$, although $${\mathbf{VV^{\prime}}} \ne {\mathbf{I}}$$.

The SNP-BLUP equations can then be re-arranged into an equivalent PCRR equation system (see Appendix):

9 $$\left[ {{\mathbf{S}}^{2} + \lambda {\mathbf{I}}} \right]{\hat{\mathbf{s}}} = {\mathbf{T}}'{\mathbf{y}}.$$

Note that ${\mathbf{T^{\prime}T}} = {\mathbf{SU^{\prime}US}} = {\mathbf{S}}^{2}$. Predictions of individual genetic effects can then be obtained as:

10 $${\hat{\mathbf{g}}} = {\mathbf{T}\hat{\mathbf{s}}} .$$

In this system of equations, there are (at most) $N$ independent effects to be estimated, rather than $k$ effects (number of loci), and both ${\mathbf{S}}^{2}$ and **I** are diagonal matrices. Hence, the entire left-hand side of the BLUP equation system is diagonal, with diagonal elements $\left( {S_{ii}^{2} + \lambda } \right)$. This equation system is extremely easy to solve, even for very large ${\mathbf{y}}$ and many genotypes and animals. The main challenge thus lies in performing SVD of matrix ${\mathbf{X}}$.

### Performing large-scale SVD analyses on genomic data

Although both population size and the number of loci can be substantial, the effective number of loci is limited by $N_{e}$, which may be rather small in farmed animal populations. According to Meuwissen et al. [15], the effective number of loci in a population is: $M_{e} = \frac{{2N_{e} L}}{{log\left( {2N_{e} } \right)}}$, where $L$ is the genome length in Morgans. For example, for a population of $N_{e} = 200$ and $L = 20$, $M_{e} = 1335$, i.e. about 67 effective loci per Morgan. This can be explained by genomic data coming from larger haplotype blocks with restricted recombination, and a reduced number of PC can thus explain all or nearly all genetic variation, even for very large populations, when $N_{e}$ is limited (the smallest PC may actually capture genotyping errors or extremely rare alleles). Still, computing a low-rank approximation of ${\mathbf{X}}$ through SVD of the entire genotype dataset can be computationally very demanding for large $N$ and $k$. One possibility is to perform SVD on a subset of the individuals, which will be referred to as the core sample, equivalent to the core sample of the APY algorithm [4], and use the results for reduced-rank approximation of the entire genomic dataset. The core sample should be representative of the population and sufficiently large such that all or nearly all genetic variation is captured, but at the same time be restricted to a computationally manageable size. More specifically, a reduced matrix, e.g. $n$ rows (individuals) of the genotype matrix ${\mathbf{X}}$ are extracted, resulting in the matrix:

11 $${\mathbf{X}}_{n} = {\mathbf{U}}_{n} {\mathbf{S}}_{n} {\mathbf{V}}_{n}^{'} .$$

For a population with limited $M_{e}$, it is expected that a representative and moderately sized core sample would span nearly all genetic variation in the population. Hence, for increasing $n$, the most important eigenvectors of ${\mathbf{X}}_{n}^{'} {\mathbf{X}}_{n}$ will approach the most important eigenvectors of the entire ${\mathbf{X}}'{\mathbf{X}}$, i.e. the first few columns in ${\mathbf{V}}_{n}$ likely approach the first few columns in ${\mathbf{V}}$. Hence, ${\mathbf{V}}_{n}$ can be used to approximate the scores for the non-core animals. In the case where SVD is performed on the entire dataset, the score matrix is: ${\mathbf{T}} = {\mathbf{XV}}\left( { = {\mathbf{USV^{\prime}V}} = {\mathbf{US}}} \right)$. For a reduced-dimension model, the score matrix is: ${\mathbf{T}}_{q} = {\mathbf{XV}}_{q}$, where ${\mathbf{V}}_{q}$ includes the first $q$ eigenvectors of ${\mathbf{V}}$. As now SVD is performed on a smaller core sample, the reduced-dimension score matrix can be estimated by replacing ${\mathbf{V}}_{q}$ with ${\mathbf{V}}_{nq}$ (i.e. the first $q$ eigenvectors of ${\mathbf{V}}_{n}$):

12 $${\mathbf{C}} = {\mathbf{XV}}_{nq} .$$

The model can now be written as:

13 $${\mathbf{y}} = {\mathbf{Cs}} + {\mathbf{e}},$$

and the PCRR equation system becomes:

14 $$\left[ {{\mathbf{C^{\prime}C}} + \lambda {\mathbf{I}}} \right]{\hat{\mathbf{s}}} = {\mathbf{C}}'{\mathbf{y}}.$$

Now, ${\hat{\mathbf{s}}} = {\mathbf{V}}_{nq} '{\hat{\mathbf{b}}}$ (i.e. ${\mathbf{V}}_{nq}$ has replaced ${\mathbf{V}}_{q}$ from the entire population). Note that ${\mathbf{C}}\varvec{'}{\mathbf{C}}$ is not a diagonal matrix. The dimension of this equation system (genomic effects) is the number of chosen components (based on the core sample), $q$ $\left( { \le n} \right)$. Hence, given that an SVD can be performed on the $n \times k$ genomic dataset of the core sample, a direct solution to the (maximum) $n \times n$ PCRR equation system would be straightforward.

Alternatively, dimensionality reduction and SVD of the entire genomic data set can be performed in three steps: (1) SVD on genomic data of the core sub-sample; (2) dimensionality reduction of the entire genomic data ${\mathbf{X}}$ set using Eq. (12), resulting in the reduced-dimension matrix ${\mathbf{C}}$; and (3) SVD of ${\mathbf{C}}$ (without further dimensionality reduction), resulting in a score matrix ${\hat{\mathbf{T}}}$ of the entire genomic data set ${\mathbf{X}}$. Hence:

15 $${\mathbf{C}} = {\mathbf{U}}_{{\mathbf{C}}} {\mathbf{S}}_{{\mathbf{C}}} {\mathbf{V}}_{{\mathbf{C}}}^{'} = {\hat{\mathbf{T}}\mathbf{V}}_{{\mathbf{C}}}^{\prime} .$$

Now: $${\mathbf{X}} \approx {\mathbf{CV}}_{nq} \varvec{'} = {\hat{\mathbf{T}}\mathbf{V}}_{{\mathbf{C}}}^{\prime} {\mathbf{V}}_{{{\mathbf{nq}}}} \varvec{'} = {\hat{\mathbf{T}}{\hat{\mathbf{V}}}}\varvec{'}$$.

The model is now:

16 $${\mathbf{y}} = {\hat{\mathbf{T}}\mathbf{t}} + {\mathbf{e}}.$$

Here, ${\hat{\mathbf{t}}} = {\mathbf{V}}_{{\mathbf{C}}}^{'} {\hat{\mathbf{s}}} = {\mathbf{V}}_{{\mathbf{C}}}^{'} {\mathbf{V}}_{nq}^{'} {\hat{\mathbf{b}}} = {\hat{\mathbf{V}}}\varvec{'}{\hat{\mathbf{b}}}$. Note that ${\hat{\mathbf{T}}}$ has the same dimension as ${\mathbf{C}}$, but ${\hat{\mathbf{T}}}\varvec{'}{\hat{\mathbf{T}}} = {\mathbf{S}}_{{\mathbf{C}}}^{2}$ (diagonal) and ${\hat{\mathbf{V}}}\varvec{'}{\hat{\mathbf{V}}} = {\mathbf{I}}$. The PCRR equation system is thus:

17 $$\left[ {{\mathbf{S}}_{{\mathbf{C}}}^{2} + \lambda {\mathbf{I}}} \right]{\hat{\mathbf{t}}} = {\hat{\mathbf{T}}}'{\mathbf{y}},$$

for which the coefficient matrix is diagonal, making the equation system easy to solve.

A small numerical example illustrates the method. Consider the genotypes of five individuals (the four given in the earlier example and an additional animal): $${\mathbf{X}} = \left[ {\begin{array}{*{20}c} 0 & 1 & 0 & 1 & 0 \\ 1 & { - \,1} & { - \,1} & 1 & 0 \\ { - \,1} & 1 & 1 & { - \,1} & 0 \\ 0 & 0 & { - \,1} & { - \,1} & 1 \\ 0 & 1 & 0 & 1 & 0 \\ \end{array} } \right]$$. This centered genotype matrix still has rank 3 and, thus, there is room for dimension reduction. The genotype of the last individual is identical to the first individual, and thus we consider the first four individuals as core sample and use this in SVD (keeping the first three components):

$${\mathbf{X}}_{n} = \left[ {\begin{array}{*{20}c} 0 & 1 & 0 & 1 & 0 \\ 1 & { - \,1} & { - \,1} & 1 & 0 \\ { - \,1} & 1 & 1 & { - \,1} & 0 \\ 0 & 0 & { - \,1} & { - \,1} & 1 \\ \end{array} } \right] = {\mathbf{U}}_{n} {\mathbf{S}}_{n} {\mathbf{V}}_{n}^{\prime}$$

$${\mathbf{C}} = {\mathbf{XV}}_{n3} = \left[ {\begin{array}{*{20}c} {0.00} & {1.00} & { - \,1.00} \\ {2.00} & {0.00} & {0.00} \\ { - \,2.00} & {0.00} & {0.00} \\ {0.00} & { - \,1.62} & { - \,0.62} \\ {0.00} & {1.00} & { - \,1.00} \\ \end{array} } \right].$$

Matrix ${\mathbf{C}}$ can be used directly in PCRR. Assume that the five individuals have the following phenotypes (assuming no fixed effects): $${\mathbf{y}} = \left[ {\begin{array}{*{20}c} {\begin{array}{*{20}c} { - \,0.5} \\ { - \,0.5} \\ \end{array} } \\ {\begin{array}{*{20}c} {0.0} \\ {1.0} \\ \end{array} } \\ { - \,0.7} \\ \end{array} } \right]$$ and $\lambda = 1$. Then, solving the equation system: $\left[ {{\mathbf{C^{\prime}C}} + \lambda {\mathbf{I}}} \right]{\hat{\mathbf{s}}} = {\mathbf{C}^{\prime}\mathbf{y}}$, yields $${\hat{\mathbf{s}}} = \left[ {\begin{array}{*{20}c} { - \,0.111} \\ { - \,0.497} \\ {0.025} \\ \end{array} } \right]$$ and $${\hat{\mathbf{g}}} = {\mathbf{C}}{\hat{\mathbf{s}}} = \left[ {\begin{array}{*{20}c} { - \,0.522} \\ { - \,0.222} \\ {0.222} \\ {0.789} \\ { - \,0.522} \\ \end{array} } \right]$$.

Note that ${\mathbf{C}}'{\mathbf{C}}$ is not a diagonal matrix. Alternatively, a second-stage SVD can be performed, giving ${\mathbf{C}} = {\mathbf{U}}_{{\mathbf{C}}} {\mathbf{S}}_{{\mathbf{C}}} {\mathbf{V}}_{{\mathbf{C}}}^{'} = {\hat{\mathbf{T}}\mathbf{V}}_{{\mathbf{C}}}^{\prime}$. Now: $${\hat{\mathbf{T}}} = {\mathbf{U}}_{{\mathbf{C}}} {\mathbf{S}}_{{\mathbf{C}}} = \left[ {\begin{array}{*{20}c} {0.00} & {1.29} & { - \,0.57} & {0.00} \\ {2.00} & {0.00} & {0.00} & {0.00} \\ { - \,2.00} & {0.00} & {0.00} & {0.00} \\ {0.00} & { - \,1.29} & { - \,1.15} & {0.00} \\ {0.00} & {1.29} & { - \,0.57} & {0.00} \\ \end{array} } \right]$$.

Here, ${\hat{\mathbf{T}}}\varvec{'}{\hat{\mathbf{T}}} = {\mathbf{S}}_{{\mathbf{C}}}^{2}$ (diagonal) and solving the equation system $\left[ {{\mathbf{S}}_{{\mathbf{C}}}^{2} + \lambda {\mathbf{I}}} \right]{\hat{\mathbf{t}}} = {\hat{\mathbf{T}}}^{'} {\mathbf{y}}$ yields $${\hat{\mathbf{t}}} = \left[ {\begin{array}{*{20}c} {0.111} \\ {0.473} \\ { - \,0.154} \\ \end{array} } \right]$$, and $${\hat{\mathbf{g}}} = {\hat{\mathbf{T}}\hat{\mathbf{t}}} = \left[{\begin{array}{*{20}c} { - \,0.522} \\ { - \,0.222} \\ {0.222} \\ {0.789} \\ { - \,0.522} \\ \end{array} } \right]$$, i.e. exactly the same animal solutions as above.

### Performing SVD in parallel on genome segments

The SVD can be performed independently (in parallel) on different genome segments (in this case, chromosomes). This implies that different (but not necessarily fully independent) sets of PC are chosen for each segment. For the core sample, the economy-sized SVD of chromosome $i$ is thus:

18 $${\mathbf{X}}_{in} = {\mathbf{U}}_{in} {\mathbf{S}}_{in} {\mathbf{V}}_{in}^{'} ,$$

19 $${\mathbf{T}}_{in} = {\mathbf{U}}_{in} {\mathbf{S}}_{in} .$$

As above, the approximated score matrix ${\hat{\mathbf{T}}}$ of ${\mathbf{X}}$ can be computed in three steps: (1) perform chromosome-wise SVD on a core sample of genomic data for each chromosome (same core individuals for all chromosomes); (2) compute chromosome-specific reduced rank ${\mathbf{C}}_{i} = {\mathbf{X}}_{i} {\mathbf{V}}_{inq}$ for all individuals (core and non-core) and concatenate these into $${\mathbf{C}} = \left[ {\begin{array}{*{20}l} {\begin{array}{*{20}c} {{\mathbf{C}}_{1} } & {{\mathbf{C}}_{2} } \\ \end{array} } & {\begin{array}{*{20}c} \ldots & {{\mathbf{C}}_{c} } \\ \end{array} } \\ \end{array} } \right]$$; and (3) perform SVD of ${\mathbf{C}} = {\mathbf{U}}_{{\mathbf{C}}} {\mathbf{S}}_{{\mathbf{C}}} {\mathbf{V}}_{{\mathbf{C}}} \varvec{'}$ and compute the reduced dimension score matrix ${\hat{\mathbf{T}}} = {\mathbf{U}}_{{\mathbf{C}}} {\mathbf{S}}_{{\mathbf{C}}}$ (without further rank reduction).

The entire genotype matrix across all chromosomes can then be approximated as:

20 $${\mathbf{X}} \approx {\mathbf{C}}\left[ {\begin{array}{*{20}c} {{\mathbf{V}}_{1nq} \varvec{'}} & 0 & 0 \\ 0 & \ldots & 0 \\ 0 & 0 & {{\mathbf{V}}_{cnq} \varvec{'}} \\ \end{array} } \right] = {\hat{\mathbf{T}}\mathbf{V}}_{{\mathbf{C}}}^{'} \left[ {\begin{array}{*{20}c} {{\mathbf{V}}_{1nq} \varvec{'}} & 0 & 0 \\ 0 & \ldots & 0 \\ 0 & 0 & {{\mathbf{V}}_{cnq} \varvec{'}} \\ \end{array} } \right] = {{\hat{\mathbf{T}}\hat{\mathbf{V}}}}.$$

The model and equation system are then as described above (Eqs. 16 and 17). As above, matrix ${\mathbf{C}}$ can also be used directly in PCRR, although the mixed model coefficient matrix may be dense (but of reduced dimensionality).

For each chromosome, the effective number of segregating loci is much smaller than for the whole genome, implying that fewer PC ($< n$) will be needed per chromosome than for the whole genome. The total number of chosen PC (at most $n \times c$, where $c$ is the number of chromosomes) is $\sum q_{i}$, where $q_{i}$ is the number of chosen PC for chromosome $i$. Still, since SVD of the core sample genomic data is performed chromosome-wise, the final number of chosen PC may potentially exceed the number of animals in the core subpopulation. This implies that genetic variation of the core and non-core subpopulations is assumed to be explained by a limited number of common components (i.e. haplotype blocks), and that the number of components that segregate in the core may be larger than the number of core individuals. In contrast, the APY algorithm assumes that all genetic variation is explained by the additive genetic effects of the core individuals, rather than by the haplotype blocks that segregate among those individuals.

### Principal component based algorithm for inverting the GRM (PCIG)

Single-step genomic analyses are widely used in the analysis of real data. As mentioned earlier, the (original) single-step equation system requires the inverse of the GRM $\left( {{\mathbf{G}}^{ - 1} } \right)$ to be computed prior to analysis. If inversion is done by “brute force”, large-scale analyses that potentially include millions of genotyped animals will be virtually impossible to perform. However, in the following section we describe how the GRM for such data can be effectively approximated through SVD techniques and how the exact inverse of an approximated GRM can be obtained.

If SVD of ${\mathbf{C}}$ can be performed as described above: ${\mathbf{X}} \approx {\hat{\mathbf{T}}\hat{\mathbf{V}}}'$. A PC-based GRM then is:

21 $${\mathbf{G}} = \frac{1}{\rho } \cdot {\mathbf{XX^{\prime}}} \approx \frac{1}{\rho } \cdot {\hat{\mathbf{T}}\hat{\mathbf{V}}}'{\hat{\mathbf{V}}\hat{\mathbf{T}}}^{'} = \frac{1}{\rho } \cdot {\hat{\mathbf{T}}\hat{\mathbf{T}}}',$$

where $\rho = 2{\mathbf{p}}'\left( {1 - {\mathbf{p}}} \right)$, with ${\mathbf{p}}$ being a vector of SNP allele frequencies in the population. An actual inverse of ${\mathbf{G}}$ may not exist, as ${\mathbf{XX}^{\prime}}$ may not have full rank (even with very dense SNP data), while a reduced-rank ${\hat{\mathbf{T}}\hat{\mathbf{T}}}\varvec{'}$ (rank $< N$) is never invertible. As above, this problem can be circumvented by replacing ${\mathbf{G}}$ with ${\tilde{\mathbf{G}}} = \rho {\hat{\mathbf{T}}\hat{\mathbf{T}}}^{'} + {\mathbf{I}}\theta = \frac{1}{\rho }\left( {{\hat{\mathbf{T}}\hat{\mathbf{T}}}}^{'} + {\mathbf{I}}\rho \theta \right)$, where $\theta$ is a small value (e.g. 10^−3^) to ensure that ${\tilde{\mathbf{G}}}$ is positive definite and thus can be inverted. Using the Woodbury formula [10], the exact inverse of ${\tilde{\mathbf{G}}}$ is:

22 $$\begin{aligned} {\tilde{\mathbf{G}}}^{ - 1} & = \rho \left( {{\hat{\mathbf{T}}\hat{\mathbf{T}}}}\varvec{'} + {\mathbf{I}}\rho \theta \right)^{ - 1} \\ & = \rho \left( {{\mathbf{I}}\rho^{ - 1} \theta^{ - 1} - {\mathbf{I}}\rho^{ - 1} \theta^{ - 1} {\hat{\mathbf{T}}}\left( {{\mathbf{I}}_{{\mathbf{p}}} + {\hat{\mathbf{T}}}'{\mathbf{I}}\rho^{ - 1} \theta^{ - 1} {\hat{\mathbf{T}}}} \right)^{ - 1} {\hat{\mathbf{T}}}'{\mathbf{I}}\rho^{ - 1} \theta^{ - 1} } \right) \\ & = \frac{1}{\theta }\left( {{\mathbf{I}} - {\hat{\mathbf{T}}}\left( {{\mathbf{S}}_{{\mathbf{C}}}^{2} + {\mathbf{I}}_{{\mathbf{p}}} \rho \theta } \right)^{ - 1} {\hat{\mathbf{T}}}} \right), \\ \end{aligned}$$

where ${\mathbf{I}}_{{\mathbf{p}}}$ is an identity matrix of dimension $\sum q_{i}$ (number of chosen PC summed over all chromosomes). The only matrix that needs to be inverted explicitly is $\left( {{\mathbf{S}}_{{\mathbf{C}}}^{2} + {\mathbf{I}}_{{\mathbf{p}}} \rho \theta } \right)$, which is diagonal. Hence, given that ${\mathbf{S}}_{{\mathbf{C}}}^{2}$ and ${\hat{\mathbf{T}}}$ are available, computing ${\tilde{\mathbf{G}}}^{ - 1}$ is not very demanding. Furthermore, the inverse relationships can be computed row by row as:

$${\tilde{\mathbf{G}}}_{\varvec{i}}^{ - 1} = \frac{1}{\theta }\left( {{\mathbf{I}}_{\varvec{i}} - {\hat{\mathbf{T}}}_{\varvec{i}} \left( {{\mathbf{S}}_{{\mathbf{C}}}^{2} + {\mathbf{I}}_{{\mathbf{p}}} \rho \theta } \right)^{ - 1} {\hat{\mathbf{T}}}'} \right).$$

The above inverse of GRM requires an SVD of the ${\mathbf{C}}$ matrix (as described in the stepwise procedures above). However, since ${\mathbf{CC^{\prime}}} = {\hat{\mathbf{T}}\mathbf{V}}_{{\mathbf{c}}} \varvec{'}{\mathbf{V}}_{{\mathbf{c}}} {\hat{\mathbf{T}}}\varvec{'} = {\hat{\mathbf{T}}\hat{\mathbf{T}}}\varvec{'}$, the above inverse of the GRM can also be computed as:

23 $$\begin{aligned} {\tilde{\mathbf{G}}}^{ - 1} & = \rho \left( {{\mathbf{CC^{\prime}}} + {\mathbf{I}}\rho \theta } \right)^{ - 1} \\ & = \rho \left( {{\mathbf{I}}\rho^{ - 1} \theta^{ - 1} - {\mathbf{I}}\rho^{ - 1} \theta^{ - 1} {\mathbf{C}}\left( {{\mathbf{I}}_{{\mathbf{p}}} + {\mathbf{C^{\prime}I}}\rho^{ - 1} \theta^{ - 1} {\mathbf{C}}} \right)^{ - 1} {\mathbf{C^{\prime}I}}\rho^{ - 1} \theta^{ - 1} } \right) \\ & = \frac{1}{\theta }\left( {{\mathbf{I}} - {\mathbf{C}}\left( {{\mathbf{C^{\prime}C}} + {\mathbf{I}}_{{\mathbf{p}}} \rho \theta } \right)^{ - 1} {\mathbf{C}}'} \right). \\ \end{aligned}$$

Thus, the only explicit inverse needed here is $\left( {{\mathbf{C^{\prime}C}} + {\mathbf{I}}_{{\mathbf{p}}} \rho \theta } \right)^{ - 1}$, which is of full rank and has dimension $\sum q_{i}$. For example, $\sum q_{i} \le$ 10,000 components may be sufficient to describe essentially all genetic variation, even for a large genotyped population if it has limited $N_{e}$. Under these assumptions, an inverse of GRM can be computed for any number of genotyped individuals.

### QR-based algorithm for inverting GRM (QRIG)

Fernando et al. [5] suggested a QR decomposition of the ${\mathbf{X}}$ matrix, which is generally faster than SVD. A QR decomposition of matrix ${\mathbf{X}}_{n} \varvec{'}$, of dimension $k \times n$, with $n < k$, is:

24 $$\begin{aligned} {\mathbf{X}}_{n} ' & = {\mathbf{Q}}_{n} {\mathbf{R}}_{n} \\ {\text{i}} . {\text{e}}.\quad {\mathbf{X}}_{n} & = {\mathbf{R}}_{n} \varvec{'}{\mathbf{Q}}_{n} \varvec{'} \\ \end{aligned} ,$$

where ${\mathbf{Q}}_{n}$ is a $k \times n$ matrix with orthogonal columns (i.e. ${\mathbf{Q}}_{n}^{'} {\mathbf{Q}}_{n} = {\mathbf{I}}, {\mathbf{Q}}_{n} {\mathbf{Q}}_{n} ' \ne {\mathbf{I}}$), while ${\mathbf{R}}_{n}$ is a $n \times n$ upper triangular matrix. Furthermore, ${\mathbf{R}}_{n}^{\varvec{'}} = {\mathbf{X}}_{n} {\mathbf{Q}}_{n}$. The genomic relationship matrix for the core sample is:

25 $${\mathbf{G}}_{n} = \frac{1}{\rho } \cdot {\mathbf{X}}_{n} {\mathbf{X}}_{n}^{'} = \frac{1}{\rho } \cdot {\mathbf{R}}_{n}^{'} {\mathbf{Q}}_{n}^{'} {\mathbf{Q}}_{n} {\mathbf{R}}_{n} = \frac{1}{\rho } \cdot {\mathbf{R}}_{n} '{\mathbf{R}}_{n} .$$

As in the APY algorithm, this method assumes that (nearly) all genetic variation is captured by the additive genetic effects of individuals in the core sample. For the entire dataset ${\mathbf{X}}$ (sorted such that the core sample comes first), this implies that:

26 $${\mathbf{X}} = {\mathbf{R^{\prime}Q^{\prime}}} \approx \left[ {\begin{array}{*{20}c} {{\mathbf{R}}_{n} '} & 0 \\ {{\hat{\mathbf{R}}}_{ - n} '} & 0 \\ \end{array} } \right]\left[ {\begin{array}{*{20}c} {{\mathbf{Q}}_{n} '} & 0 \\ 0 & 0 \\ \end{array} } \right] = {\hat{\mathbf{R}}}\varvec{'}{\mathbf{Q}}_{n} \varvec{',}$$

where $${\hat{\mathbf{R}}}\varvec{'} = \left[ {\begin{array}{*{20}c} {{\mathbf{R}}_{n} '} \\ {{\hat{\mathbf{R}}}_{ - n} '} \\ \end{array} } \right]$$, ${\hat{\mathbf{R}}}_{ - n}^{'} = {\mathbf{X}}_{ - n} {\mathbf{Q}}_{n}$, and ${\mathbf{X}}_{{ - {\text{n}}}}$ is the genotype matrix of all non-core individuals. The GRM can thus be approximated as:

27 $${\mathbf{G}} \approx \frac{1}{\rho } \cdot {\hat{\mathbf{R}}}\varvec{'}{\mathbf{Q}}_{n} \varvec{'}{\mathbf{Q}}_{n} {\hat{\mathbf{R}}} = \frac{1}{\rho } \cdot {\hat{\mathbf{R}}}\varvec{'}{\hat{\mathbf{R}}},$$

where ${\hat{\mathbf{R}}}$ is a $n \times N$ matrix, which is considerably smaller than the original ${\mathbf{X}}$ ($N \times k$). This approach is equivalent to strategy IV of Fernando et al. [5] (except that core animals are assumed to explain nearly all genomic variation rather than all genomic variation exactly). Here, genotypes of all animals are expressed as linear functions of genotypes of a reduced set of animals (rows in the genotype matrix). In their case, this result was used to compute a reduced set of components. Here, instead we use ${\hat{\mathbf{R}}}$ to compute a QR-based inverse of GRM (QRIG) as:

28 $${\tilde{\mathbf{G}}}^{ - 1} = \rho \left( {{\hat{\mathbf{R}}}\varvec{'}{\hat{\mathbf{R}}} + {\mathbf{I}}\rho \theta } \right)^{ - 1} = \frac{1}{\theta }\left( {{\mathbf{I}} - {\hat{\mathbf{R}}}\left( {{\hat{\mathbf{R}}}^{'} {\hat{\mathbf{R}}} + {\mathbf{I}}_{p} \rho \theta } \right)^{ - 1} {\hat{\mathbf{R}}}'} \right).$$

Thus, the only part that needs to be inverted explicitly is the $n \times n$ matrix $\left( {{\hat{\mathbf{R}}}^{'} {\hat{\mathbf{R}}} + {\mathbf{I}}_{p} \rho \theta } \right)$. The QR factorization can, as for SVD, be parallelized through chromosome-wise factorizations. Then, an overall QR is performed on the combined ${\mathbf{R}}$ matrix:

29 $$\left[ {\begin{array}{*{20}c} {\begin{array}{*{20}c} {{\mathbf{R}}_{1n} } \\ {{\mathbf{R}}_{2n} } \\ \end{array} } \\ {\begin{array}{*{20}c} \ldots \\ {{\mathbf{R}}_{in} } \\ \end{array} } \\ \end{array} } \right] = {\mathbf{Q}}_{n} {\mathbf{R}}_{n} ,$$

where ${\mathbf{R}}_{in}$ is an ${\mathbf{R}}$ matrix that is obtained from QR factorization of the genomic data on chromosome $i$ and ${\mathbf{R}}_{n}$ is a genome-wide matrix. The QRIG algorithm is well suited for reduced-rank approximations down to the size of the core sample. However, reducing rank below the size of the core sample would not be optimal (e.g. in chromosome-wise analysis), as this implies a reduction in the size of the core sample. For such situations, the PCIG approach is more appropriate because this method uses all available information in the core sample to estimate a reduced number of contributing components.

### Weighted genomic relationship matrix

As in MEM, different loci can be given different relative weights in the genomic animal model by weighting SNPs differently in the calculation of the GRM, as:

30 $${\mathbf{G}} = \frac{1}{\rho }{\mathbf{XDX^{\prime}}} = \frac{1}{\rho }{\mathbf{USV}}'{\mathbf{DVSU}}' = \frac{1}{\rho }{\mathbf{TFT}}',$$

where ${\mathbf{D}}$ is a diagonal matrix of locus weights (proportional to the variance of the effect of each locus) and $\rho = 2{\mathbf{p}}'{\mathbf{D}}\left( {1 - {\mathbf{p}}} \right)$, with ${\mathbf{p}}$ being a vector of allele frequencies in the population, and ${\mathbf{F}} = {\mathbf{V}}'{\mathbf{DV}}$. In the simplest case, i.e. using VanRaden Method 2 [16], elements of ${\mathbf{D}}$ are: $D_{i} = \frac{1}{{2p_{i} \left( {1 - p_{i} } \right)}}$. The GRM can then be approximated as:

31 $${\mathbf{G}} \approx \frac{1}{\rho }{\mathbf{CV}}_{nq} '{\mathbf{DV}}_{nq} {\mathbf{C^{\prime}}} = \frac{1}{\rho }{\mathbf{CF}}_{n} {\mathbf{C^{\prime}}},$$

where ${\mathbf{F}}_{n} = {\mathbf{V}}_{nq} '{\mathbf{DV}}_{nq}$, i.e. a symmetric matrix with dimension equal to the number of chosen components. The exact inverse of the approximated genomic relationship matrix is:

32 $$\begin{aligned} {\tilde{\mathbf{G}}}^{ - 1} & = \rho \left( {{\mathbf{CF}}_{n} {\mathbf{C^{\prime}}} + {\mathbf{I}}\rho \theta } \right)^{ - 1} \\ & = \rho \left( {{\mathbf{I}}\rho^{ - 1} \theta^{ - 1} - {\mathbf{I}}\rho^{ - 1} \theta^{ - 1} {\mathbf{C}}\left( {{\mathbf{F}}_{n}^{ - 1} + {\mathbf{C^{\prime}I}}\rho^{ - 1} \theta^{ - 1} {\mathbf{C}}} \right)^{ - 1} {\mathbf{C^{\prime}I}}\rho^{ - 1} \theta^{ - 1} } \right) \\ & = \frac{1}{\theta }\left( {{\mathbf{I}} - {\mathbf{C}}\left( {{\mathbf{C^{\prime}C}} + {\mathbf{F}}_{n}^{ - 1} \rho \theta } \right)^{ - 1} {\mathbf{C^{\prime}}}} \right). \\ \end{aligned}$$

Using this method, a weighted genomic relationship matrix can be used even for single-step animal models.

### Simulation study

A simulation study was performed to verify the reduced-rank approximation of genomic data. The simulated species had 20 chromosomes of 1 Morgan each. Simulation of sequence data followed the approach of Meuwissen and Goddard [17], except that their scaling argument was not applied here, in order not to scale down the computations. The historical effective population size was 1000, which also reflects its actual size, since simulation of new generations followed Wright’s idealized population structure. To create LD and mutation-drift equilibria, the historical population was simulated for 10,000 generations. The per meiosis and per base pair mutation rate was 10^−8^ and mutations followed the infinite sites model [18]. After the initial 10,000 generations, $N_{e}$ was reduced to 100 over 10 generations to mimic a livestock population. In the last generation, 10,000 animals were generated and their genotypes and phenotypes were used in genetic analysis. The total number of segregating loci in generation 10,000 was 531,836, of which about half (279,504) were still segregating in the last generation (generation 10,010). Per chromosome, 200 SNPs with a minor allele frequency higher than 0.01 were randomly sampled as causative SNPs, i.e. 4000 causative SNPs in total. Genotypes were standardized to $\frac{{ - 2p_{j} }}{{\sqrt {2p_{j} \left( {1 - p_{j} } \right)} }}$, $\frac{{1 - 2p_{j} }}{{\sqrt {2p_{j} \left( {1 - p_{j} } \right)} }}$ and $\frac{{2 - 2p_{j} }}{{\sqrt {2p_{j} \left( {1 - p_{j} } \right)} }}$ for the genotypes ‘0 0’, ‘0 1’ and ‘1 1’, respectively, where $p_{j}$ is the frequency of the ‘1’ allele, and collected in the genotype matrix ${\mathbf{X}}$. True genetic values of the animals were obtained as:

33 $${\mathbf{TBV}} =\upalpha{\mathbf{Xb}},$$

where ${\mathbf{b}}$ is a (531,836 × 1) vector, including 4000 quantitative trait loci (QTL) (SNPs that were declared as QTL) effects, which were sampled from a normal distribution, and effects of non-causative SNPs set to 0. All QTL effects were scaled by $\upalpha$ such that total additive genetic variance in generation 10,001 was $\sigma_{g}^{2} = 1.0$. Residual environmental effects were sampled from $N\left( {\mathbf{{0}},{\mathbf{I}}\sigma_{e}^{2} } \right)$, with $\sigma_{e}^{2}$ set such that heritability was 0.25, 0.50 or 0.90. No fixed effects were simulated. The resulting dataset was analyzed with several statistical models:

1. Ordinary GBLUP
  $${\mathbf{y}} = {\mathbf{1}}\mu + {\mathbf{Zg}} + {\mathbf{e}},$$
  $${\mathbf{g}} \sim N\left( {0,{\mathbf{G}}\sigma_{g}^{2} } \right).$$
2. Reduced-rank PCRR (chromosome-wise SVD)
  $${\mathbf{y}} = {\mathbf{1}}\mu + {\hat{\mathbf{T}}\mathbf{s}} + {\mathbf{e}},$$
  $${\mathbf{s}} \sim N\left( {{\mathbf{0}},{\mathbf{I}}\sigma_{m}^{2} } \right),$$
  $${\mathbf{g}} = {\hat{\mathbf{T}}\mathbf{s}}.$$
3. GBLUP using reduced-dimension approximations of GRM
  - Chromosome-wise SVD (PCIG-C)
  - Genome-wide SVD (PCIG-G)
  - QR-based (genome-wide)
  - APY (genome-wide)

$${\mathbf{y}} = {\mathbf{1}}\mu + {\mathbf{Zg}} + {\mathbf{e}},$$

$${\mathbf{g}} \sim N\left( {{\mathbf{0}},{\tilde{\mathbf{G}}}\sigma_{g}^{2} } \right),$$

where ${\tilde{\mathbf{G}}}$ is an approximation of ${\mathbf{G}}$, using the PCIG-G, PCIG-C, QRIG, or APY algorithms.

The chromosome-wise SVD (PCRR or PCIG-C) was performed independently for each chromosome based on a core sample of 500, 1000 or 2000 individuals. For each chromosome, the number of components was set such that > 99% of the chromosome-specific genomic variation (in the core) was explained by the chosen PC. These PC were then used to compute ${\hat{\mathbf{T}}}$. For the PCIG-G, an economy-sized SVD was performed across all chromosomes for the core sample (500 to 2000 individuals) and, thus, the final number of components was equal to the core sample size. The QR-based algorithm was based on all genotypes of the core sample, while the APY algorithm was based on genomic relationships of core sample individuals.

All models and algorithms were compared based on their accuracy of predicting the true breeding values of validation animals that had masked phenotypes. Validation animals were randomly sampled among non-core animals (with a probability of 10%).

Data preparation and statistical analyses were performed using Julia software scripts (http://julialang.org/). All solutions were obtained by solving the mixed model equations directly.

### Real data analysis

The PCIG-C and APY algorithms were also used in a single-step multi-trait genomic evaluation of a real dataset, which was comprised of data from the Irish beef cattle carcass evaluation and included 8.33 million animals with records on nine traits. The model used was identical (excluding genetic groups) to the standard Irish beef cattle evaluation model [19]. There were 13.35 million animals in the pedigree, of which 163,277 were genotyped. Genotyping was done by using the Illumina Bovine SNP50 Bead Chip (Illumina, San Diego, USA), of which 54,620 SNPs on 29 autosomes were included in the analysis (after quality edits). The population was heterogeneous and included genotypes of animals from 41 breeds. Hence, the dataset was challenging in the sense that a large core sample was needed to capture genetic variation in all breeds. For PCIG-C, the number of components per chromosome was set such that it explained a given percentage (from 90 to 95%) of the chromosome-specific genomic variation and core sample sizes were 30,000 to 50,000. The resulting estimated breeding values (EBV) using the PCIG-C and APY inverse GRM were compared with the original EBV based on direct inversion of $\left( {{\mathbf{G}} + {\mathbf{I}}\theta } \right)$.

The analysis was conducted using an iterative solver in the MIX99 software (http://www.luke.fi/mix99), using the preconditioned conjugate gradient method and iteration on data. A value of *θ* = 10^−3^ was added to the diagonal elements of the GRM to ensure that the matrix was positive definite.

Two simple Julia scripts are attached, demonstrating 1) how to use SVD methods to compute reduced-dimension approximations of a larger genomic data using a core sample (Additional file 1), and 2) how to combine reduced-dimension genomic data from multiple chromosomes in computation of an inverse approximated genomic relationship matrix (Additional file 2).

## Results

### Simulation study

**Fig. 1:**
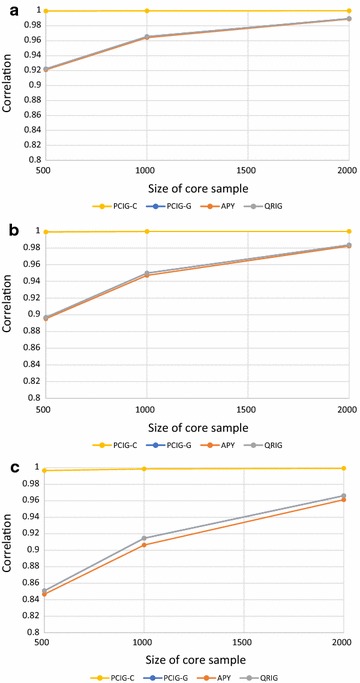
Correlations of genomic estimated breeding values for validation animals obtained using chromosome-wise PCIG (PCIG-C), genome-wise PCIG (PCIG-G), QR-based inversion (QRIG) and the APY GBLUP (APY) with those obtained using ordinary GBLUP. Based on simulated data with heritability equal to 0.25 (**a**), 0.50 (**b**), and 0.90 (**c**)

When based on the same PC, the PCIG and PCRR models are equivalent, except that for PCIG a small number is added to the diagonal elements of the GRM prior to inversion. Thus, for the simulation study, the results of PCIG and PCRR were nearly identical and only results for PCIG are shown. The correlations of the EBV from each model with the EBV of the full-dimensional GBLUP are in Fig. 1.

**Fig. 2:**
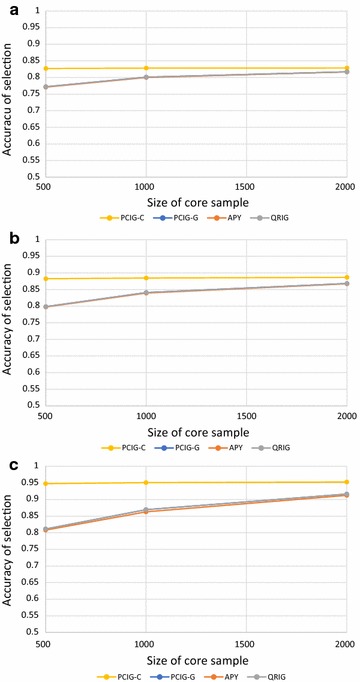
Accuracies of genomic estimated breeding values (correlation with true breeding values) for validation animals, using chromosome-wise PCIG (PCIG-C), genome-wise PCIG (PCIG-G), QR-based inversion (QRIG) and the APY GBLUP (APY). Based on simulated data with heritability equal to 0.25 (**a**), 0.50 (**b**), and 0.90 (**c**). Accuracies of the original GBLUP method were essentially identical to those from PCIG-C and are not shown

In general, across core sample sizes (500 to 2000) and heritabilities (0.25 to 0.90), the PCIG-C model resulted in very similar EBV as GBLUP, with EBV correlations ranging from 0.997 to 1.000. The results were less favorable for models PCIG-G, APY and QRIG (EBV correlations ranging from 0.847 to 0.984). Differences of the PCIG-C from the other models were largest for the lowest core sample sizes (500) and highest heritability (0.90). With respect to accuracy of selection (correlation between EBV and true breeding value), GBLUP and PCIG-C had very similar and generally higher accuracies than the other models (Fig. 2), 0.82, 0.88 and 0.95 for heritability equal to 0.25, 0.50 and 0.90, respectively.

Differences in accuracy of GBLUP/PCIG-C from the other models were largest at the lowest core samples and highest heritabilities (e.g. for a core sample of 500 and heritability 0.90, accuracy was 0.95 for GBLUP/PCIG-C vs. 0.81 for the other methods). At the lowest core sample size (500), genomic relationships were so crudely described by PCIG-G, APY and QRIG that very little information was obtained by changing the heritability from 0.25 to 0.90. At higher core sample sizes, the differences between GBLUP/PCIG-C and the other methods were smaller, but not negligible, even at core sample sizes up to 2000. As a result, PCIG-C was much more robust to core sample size and achieved comparable results to the full-dimension GBLUP, even at the smallest core sizes tested. Using PCIG-C, the average number of PC needed to capture at least 99% of the genomic variation per chromosome was 239, 298 and 340 for, respectively, 500, 1000 and 2000 animals in the core (4770, 5959 and 6795 components across all chromosomes). Hence, for this data structure, the genomic relationships could be effectively approximated with a limited number of chromosome-specific PC, even when estimated from core sample sizes down to 500 individuals.

With respect to computing time, QR decomposition (QRIG) required down to 18% less computing time than SVD (PCIG models) when applied to genomic data on single chromosomes (~ 25 k loci). The relative difference in computation time between QR and SVD was largest at smaller core samples. However, at small core samples, both methods were fast, making the relative difference in computing time less important.

### Multi-breed beef cattle data

For the real data analysis of the multiple-breed beef cattle population using PCIG-C, the results were essentially identical for core sample sizes of 30,000 and 50,000, hence only results of the latter are presented. Correlations of EBV from the PCIG-C single step models with EBV from the original GBLUP (direct inversion) model were high for all traits (0.995 to 0.999, 0.998 to 0.999, and 1.000 when the chosen components explained ≥ 85, ≥ 90 and ≥ 95% of chromosomal genomic variance, respectively). The corresponding numbers of chosen components were 30,208 (≥ 85%), 34,655 (≥ 90%), and 40,140 (≥ 95%). APY based on a core sample size of 50,000 individuals resulted in almost identical ranking of animals based on EBV as the original model (rank correlations ranging from 0.999 to 1.000), while the rank of animals based on an APY of a core sample of 30,000 individuals (corresponding in rank (i.e. no of PC) with PCIG-C ≥ 85%) had a slightly lower correlation with the rank from the original model (0.952 to 0.996). For a similar rank (~ 30,000) of the GRM (number of chosen PC in PCIG and number of core animals in APY), PCIG-C needed a smaller number of iterations to converge (1351 to 1385 vs. 1619 to 1756, for PCIG-C and APY, respectively). Computing times could not be compared directly, as these may have been influenced by other jobs running simultaneously on the computer cluster.

## Discussion

When based on the same PC, the PCRR and PCIG algorithms gave identical EBV. However, the PCIG algorithm is more flexible in that it can easily be incorporated into existing single-step genomic animal models. The results of the current study show that all reduced-dimension algorithms (PCRR, PCIG-C, PCIG-G, APY and QRIG) approach the GBLUP solutions when core sample size becomes large. However, the PCRR and PCIG-C algorithms were, by far, the most robust to reductions in core sample size. For the simulated data, the EBV were virtually identical to the EBV obtained with full-dimension GBLUP for all core sample sizes (even down to 500 individuals) and heritabilities, with correlations between EBV ranging from 0.997 to 1.000. For the other methods, accuracy of selection dropped considerably at smaller core sample sizes (500 and 1000), especially with high heritability. In the real data analysis of a multi-breed beef cattle population, core sample size was generally large and differences between methods were thus smaller, but still in the favor of PCIG-C compared with APY.

The PCIG algorithm can be used to calculate the exact inverse of an approximated GRM, even for extremely large genomic datasets that potentially contain millions of individuals and loci, using a limited number of PC per chromosome. The SVD-based PCIG-C uses all genetic data from the core sample to identify the more important PC for each chromosome, and the GRM is based on these. The method can be heavily parallelized, since SVD is performed separately for each chromosome. The number of PC needed to describe the relationship structure of a population depends on the effective number of segregating genomic segments in the population, which for large populations of limited $N_{e}$ is typically much smaller than the actual population size ($N$). After SVD, the inverse of GRM (using PCIG) can be computed easily, and potentially row- or element-wise, which gives room for further parallelization. Hence, computing time can be reduced substantially. Using iteration on data, rows of the inverse of GRM can be computed directly during iteration and, thus, the entire inverse GRM does not need to be stored explicitly. In contrast, when performing “brute force” inversion of the entire GRM, memory requirements increase quadratically and numbers of computations increase cubically with the number of animals in the population [20]. Compared with PCIG, QRIG algorithm based on QR-decomposition was slightly faster and has potential for parallelization (by chromosome). However, this model is less well suited for dimensionality reduction below core sample size (e.g. per chromosome) and is more sensitive to size of the core sample. Thus, we prefer the PCIG-C over the QRIG algorithm.

The PCIG algorithm proposed in this study is related to the APY algorithm [4], since both methods use genomic data in a core sample to approximate the (inverse) GRM of all animals. In APY, the core sample must be sufficiently small such that the inverse of the core GRM can be computed directly, and the remaining elements of the entire inverse of GRM are computed based on the inverse relationships of the core individuals and the relationships between core and non-core individuals. Furthermore, APY assumes that the non-core part of the inverse GRM is diagonal, while PCIG makes no such assumptions. Using PCIG, the GRM is approximated by a limited number of PC and by adding a small number to the diagonal elements, while the inverse of this matrix is computed by exact methods. Hence, given that the GRM can be appropriately approximated using PC estimated from the core sample, the computed inverse of GRM from PCIG will necessarily also be appropriate, which explains why solutions from reduced-rank PCIG-C were nearly identical to those obtained from full-dimension GBLUP in this study, even at the smallest core sample sizes. The genome-wide PCIG-G gave similar solutions as APY and (genome-wide) QRIG, which can be explained by the fact that the maximum number of components in genome-wide analysis is limited by the size of the core sample, while the maximum number of components in chromosome-wise PCIG-C is larger (size of core sample x number of chromosomes). For PCIG-G, APY and QRIG this is an especially limiting factor in smaller core samples, as observed with the simulated dataset, e.g. for these genome-wide methods a core size of 500 imply that the GRM is approximated by, at most, 500 “components” (PC or animal effects) while up to 10,000 PC may be used in the PCRR/PCIG-C models.

Genetic analyses based on chromosome-wise SVD of a core sample assumes that genetic variation associated with each chromosome can be explained by the chosen chromosome-specific components (i.e. haplotype blocks), and that the same components are present and responsible for genetic variation in the entire population. In contrast, the APY algorithm assumes that all genetic variation in the population is explained by the additive genetic effects of individuals in the core sample, i.e. that breeding values of non-core individuals are merely functions of breeding values of the core individuals. This implies that, if accuracies of core individuals approach unity (e.g. bulls with large daughter groups), accuracy of the entire genotyped population is also assumed to approach unity, even for newly born genotyped individuals, which is not likely to be true. Even if thousands of historical bulls with progeny are included in the core sample, the EBV of a genotyped calf is not expected to be perfect. In PCIG-C, a more realistic approach is taken, since the accuracy of non-core animals depends on the precision of the estimated effects of the underlying PC, rather than on the accuracy of the EBV of core animals. The number of underlying components may exceed the number of core individuals and, thus, a high accuracy of the EBV of core animals does not imply high accuracies for all underlying components. Thus, as the EBV of non-core animals are functions of these components, genotyped newborn animals are not necessarily assumed to be predicted accurately, even if the core animals are accurate.

In real data, population structures may be more complex and stratified. Hence, real data analyses of complex populations may require larger core samples, e.g. as in the real multi-population dataset analyzed here.

The methods used herein, only consider simple SNP-BLUP or genomic animal models, where, a priori, genetic variance is evenly distributed across the genome. However, such simplistic models likely do not use the full potential of high-density or sequence data, which may include genotypes of the causative mutations themselves. One alternative is to combine SVD techniques with methods that allow for different weighting of the SNPs in the model (i.e. approximating Bayesian variable selection models). This approach is described and evaluated in a separate study [21].

## Conclusions

We propose SVD-based methods for genomic prediction. Although SVD may be computationally demanding, the analysis can be performed on a reduced core sample of individuals and/or in parallel on different genome segments, making fast computation possible. After SVD, large-scale genomic analysis can be performed either by PC ridge regression (PCRR) or by a genomic animal model (GBLUP), with the GRM and its inverse defined by the chosen PC (PCIG). The principal component-based GRM is not of full rank but can be made invertible by adding a small number to the diagonal of the entire matrix, and its exact inverse can be easily obtained using the Woodbury formula. The inverse of the SVD-based GRM can be computed row- or element-wise, and the entire matrix does not need to be stored explicitly, e.g. when applying iteration on data. Based on simulated data, PCRR/PCIG models based on chromosome-wise SVD of genomic data from a limited core sample resulted in essentially identical solutions for the entire population as the full-dimension GBLUP model (correlations between EBV = 1.000), while other methods (genome-wide SVD, QRIG and APY) were less accurate, especially at smaller core sample sizes.

### Additional files

**Additional file 1.** How to do reduced-dimension approximation of a larger genomic data set from a specific chromosome, using SVD of data on the same chromosome in a smaller core sample.

**Additional file 2.** Combining reduced-dimension genomic data from multiple chromosomes in computation of an inverse approximated genomic relationship matrix.

## Appendix

In principal component regression, the original SNP solutions can be obtained from the principal component solutions as [14]:

$${\hat{\mathbf{b}}} = {\mathbf{V}\hat{\mathbf{s}}}.$$

Define ${\hat{{\varvec{\beta}}}} = {\mathbf{VV}}'{\hat{\mathbf{b}}}$ and ${\hat{\mathbf{s}}} = {\mathbf{V}}'{\hat{\mathbf{b}}}$. This is true, if it holds that ${\hat{{\varvec{\beta}}}} = {\hat{\mathbf{b}}}$, which we will prove now.

The columns of ${\mathbf{V}}$ are orthogonal, ${\mathbf{V}^{\prime}\mathbf{V}} = {\mathbf{I}}$, while, in many cases, ${\mathbf{VV^{\prime}}} \ne {\mathbf{I}}$ (e.g. when the number of loci exceeds the rank of the genotype matrix). As ${\mathbf{X}} = {\mathbf{USV^{\prime}}} = {\mathbf{TV^{\prime}}}$, multiplying ${\mathbf{X}}$ with ${\mathbf{VV^{\prime}}}$ yields:

$${\mathbf{X}}\left( {{\mathbf{VV^{\prime}}}} \right) = {\mathbf{TV^{\prime}VV^{\prime}}} = {\mathbf{TV^{\prime}}} = {\mathbf{X}}.$$

Hence, for any vector ${\varvec{\epsilon}}$ of length equal to number of loci, ${\mathbf{XVV^{\prime}\varvec{\epsilon }}} = {\mathbf{X\varvec{\epsilon }}}$. This implies that, ${\mathbf{X}}{\hat{{\varvec{\beta}}}} = {\mathbf{X}}{\hat{\mathbf{b}}}$, i.e. the predicted genetic effect of an animal is identical, whether based on ${\hat{{\varvec{\beta}}}}$ or ${\hat{\mathbf{b}}}$. The HMME (excluding fixed effects) using an MEM is:

$$\left[ {{\mathbf{X^{\prime}X}} + \lambda {\mathbf{I}}} \right]{\hat{\mathbf{b}}} = {\mathbf{X}}'{\mathbf{y}}.$$

Using that ${\mathbf{X}}\left( {{\mathbf{VV^{\prime}}}} \right) = {\mathbf{X}}$ and $\left( {{\mathbf{VV^{\prime}}}} \right){\mathbf{X}}' = {\mathbf{X}}'$, this equation system can easily be modified into an equation system for ${\hat{{\varvec{\beta}}}}$. First, we pre-multiply with $\left( {{\mathbf{VV^{\prime}}}} \right)$:

$$\left[ {{\mathbf{X}}'{\mathbf{X}} + \lambda {\mathbf{VV}}'} \right]{\hat{\mathbf{b}}} = {\mathbf{X}}'{\mathbf{y}},$$

as ${\hat{{\varvec{\beta}}}} = {\mathbf{VV}}'{\hat{\mathbf{b}}}$ and ${\mathbf{X}\hat{{\varvec{\beta}} }} = {\mathbf{X}\hat{\mathbf{b}}}$, the equation above is identical to:

$$\left[ {{\mathbf{X}}'{\mathbf{X}} + \lambda {\mathbf{I}}} \right]{\hat{{\varvec{\beta}}}} = {\mathbf{X}}'{\mathbf{y}}.$$

The resulting equation system is of full rank and identical to the original MEM equation system. Thus, there is a single solution vector, implying that:

$${\hat{{\varvec{\beta}}}} = {\hat{\mathbf{b}}}.$$

The SNP-BLUP equations can be reformulated into equivalent PCRR equations as follows:

$$\left[ {{\mathbf{X^{\prime}X}} + \lambda {\mathbf{I}}} \right]{\hat{\mathbf{b}}} = {\mathbf{X}}'{\mathbf{y}},$$

$$\left[ {{\mathbf{VT}}'{\mathbf{TV}}' + \lambda {\mathbf{I}}} \right]{\hat{\mathbf{b}}} = {\mathbf{VT}}'{\mathbf{y}}.$$

Pre-multiplying the equation above with ${\mathbf{V}}'$ yields:

$$\left[ {{\mathbf{T}}'{\mathbf{T}} + \lambda {\mathbf{I}}} \right]{\mathbf{V}}'{\hat{\mathbf{b}}} = {\mathbf{T}}'{\mathbf{y}}.$$

Define: $${\hat{\mathbf{s}}} = {\mathbf{V}}'{\hat{\mathbf{b}}}$$:

$$\left[ {{\mathbf{T}}'{\mathbf{T}} + \lambda {\mathbf{I}}} \right]{\hat{\mathbf{s}}} = {\mathbf{T}}'{\mathbf{y}}.$$

The PCRR and SNP-BLUP equations are thus equivalent.
